# Complete mitochondrial genome of the nurse shark *Ginglymostoma cirratum*

**DOI:** 10.1080/23802359.2016.1186514

**Published:** 2016-07-08

**Authors:** Tom Kashiwagi, Kevin M. Kingsland, Theo C. Pratt, Harold L. Pratt, Edward J. Heist

**Affiliations:** a Center for Fisheries, Aquaculture, & Aquatic Sciences, Southern Illinois University Carbondale, Carbondale, IL, USA;; b Elasmobranch Field Research Association, Summerland Key, FL, USA;; c Mote Marine Laboratory, Tropical Research Laboratory, Summerland Key, FL, USA

**Keywords:** Elasmobranch, Ginglymostomatidae, mitogenome, MiSeq, Orectolobiformes

## Abstract

We determined the complete mitochondrial genome sequence of nurse shark *Ginglymostoma cirratum.* The circular DNA of 16692 bp comprises 13 protein-coding genes, 22 transfer RNAs, 2 ribosomal RNAs, a non-coding control region and a non-coding origin of light strand replication with typical gene order of vertebrates. The nurse shark formed a well-supported clade that included whale shark *Rhincodon typus* and zebra shark *Stegostoma fasciatum* within the Orectolobiformes in a phylogenetic tree constructed with other published mitochondrial genomes of sharks.

The nurse shark *Ginglymostoma cirratum*, a member of the Family Ginglymostomatidae in the Order Orectolobiformes, is a common inshore bottom-dwelling shark, in tropical and subtropical waters in the Atlantic and eastern Pacific. Past studies using molecular markers provided insights on their population genetics (Karl et al. 2011), multiple paternity (Heist et al. 2011) and major histocompatibility complex (Ohta et al. 2000). In this study, we determined their complete mitochondrial genome, which can provide refined and better-resolved population structure information when developed and used as markers (Feutry et al. 2014; Feutry et al. 2015).

A tissue sample was taken from a shark captured and released in Dry Tortugas National Park, Florida, USA (24°37′25″ N, 82°51’53” W) on 19 June 2014 (permit no. DRTO-2013-SCI-0010). The sample is kept in the collection of EJH at Southern Illinois University Carbondale with the sample number GCI295. Genomic DNA was extracted using DNeasy kit (Qiagen, Valencia, CA). The shotgun genomic libraries were prepared with the TruSeq Nano Library construction kit from Illumina (San Diego, CA) and sequenced using MiSeq version 2 (Illumina) to acquire 2 × 250 bp paired-end reads. Resulting 6,350,144 reads were mapped to the reference mitochondrial genome of the brownbanded bamboo shark *Chiloscyllium punctatum* (GenBank accession no. NC016686) using Geneious 9.0.5 (Biomatters). The 7669 of 7745 mapped reads were then used to assemble *de novo* a circular 16,692 bp complete mitochondrial genome (GenBank accession no. KU904394) with a mean coverage of 104 (S.D. = 34, minimum =18 and maximum =202).

Annotation with Mitos (Bernt et al. 2013) and MitoAnnotator (Iwasaki et al. 2013) found that the complete mitochondrial genome of *G. cirratum* has typical vertebrate gene arrangement (Anderson et al. 1981), containing 13 protein-coding genes, 2 rRNAs, 22 tRNAs, a noncoding control region and a non-coding origin of light strand replication. Most of these genes were encoded on the H-strand, except for the ND6 gene and 8 tRNA genes encoded on the L-strand. Short overlaps (1–21 bp) at four gene junctions and intergenic spacers (1–5 bp) at 11 gene junctions were found. All tRNA genes had typical cloverleaf secondary structure.

Maximum likelihood tree (Figure 1), with 12 other species of sharks representing four orders, was constructed using PhyML (Guindon & Gascuel 2003) with GTR + I + G model from the alignment constructed using Muscle (Edgar 2004). Based on this taxon sampling, *G. cirratum* formed a clade with *Rhincodon typus* and *Stegostoma fasciatum* within a monophyletic Orectolobiformes, as expected.

**Figure 1.**
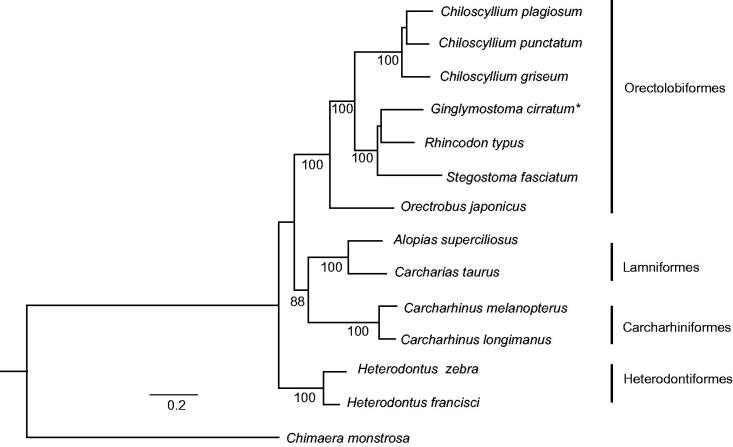
Phylogenetic tree showing *Ginglymostoma cirratum* forming a clade with *Rhincodon typus* and *Stegostoma fasciatum*. Numbers below each node represent bootstrap values from 100 replicates. GenBank accession numbers are *Chiloscyllium plagiosum* (NC_012570), *Chiloscyllium punctatum* (NC_016686), *Chiloscyllium griseum* (JQ434458), *Ginglymostoma cirratum* (KU904394), *Rhincodon typus* (KC633221), *Stegostoma fasciatum* (KU057952), *Orectrobus japonicus* (KF111729), *Alopias superciliosus* (KC757415), *Carcharias taurus* (KF569943), *Carcharhinus melanopterus* (NC_024284), *Carcharhinus longimanus* (NC_025520), *Heterodontus zebra* (KC845548), *Heterodontus francisci* (NC_003137) and *Chimaera monstrosa* (AJ310140).
